# Supplementary material for: Geographical–Historical Analysis of the Herbarium Specimens Representing the Economically Important Family Amaranthaceae (Chenopodiaceae-Amaranthaceae Clade) Collected in 1821–2022 and Preserved in the Herbarium of the Jagiellonian University in Krakow
Source: Biology (Basel). 2024 Jun 13;13(6):435. doi: 10.3390/biology13060435 (PMC11201225; doi:10.3390/biology13060435)
Supplement: Supplementary file 1 [file biology-13-00435-s001.zip › Table S2.pdf]

Table S2. A list of rare species with small ranges around the world of the *Amaranthaceae* family deposited in the KRA herbarium.

Name species	Numer of sheet in Table S1 („Scan numer")	Area of natural occurrence [1]	Threats
Achyropsis laniceps C.B.Clarke	1. UJ–KRA–P–36817–N / KRA–P–298426<br>2. UJ–KRA–P–36818–N / KRA–P–298425<br>3. UJ–KRA–P–36819–N / KRA–P–298417<br>4. UJ–KRA–P–36820–N / KRA–P–298418<br>5. UJ–KRA–P–36821–N / KRA–P–298419	Burundi, Malawi, Tanzania, Zambia, Zaïre	–
Aerva microphylla Moq.	UJ–KRA–P–36771–N / KRA–P–182950	Socotra<br>Endemic [2]	LC [3]
Aerva revoluta Balf. f.	UJ–KRA–P–36771–N / KRA–P–182950	Socotra<br>Endemic [2]	LC [3]
Amaranthus mitchellii Beneth.	1. UJ–KRA–P–36196–N / KRA–P–108664<br>2. UJ–KRA–P–36197–N / KRA–P–108663<br>3. UJ–KRA–P–36198–N / KRA–P–110784	New South Wales, Northern Territory, Queensland, South Australia, Western Australia	
Amaranthus pumilus Raf.	UJ–KRA–P–35684–N / KRA–P–184678	Connecticut, Delaware, Maryland, New York, North Carolina, Pennsylvania, Rhode I., South Carolina<br><br>Extinct: Massachusetts, New Jersey, Virginia<br><br>plant endemic to the barrier island beaches of the Atlantic coast [4,5]	different conservation protection statuses depending on the State/Provincial [6]
Anabasis eugeniae Iljin.	UJ–KRA–P–43071–N / KRA–P–277341	Iran, Transcaucasus endemic to Azerbaijan [7]	VU [8]
Atriplex australasica Moq.	UJ–KRA–P–39978–N / KRA–P–313842	New South Wales, South Australia, Tasmania, Victoria	
Atriplex californica Moq.	UJ–KRA–P–39992–N / KRA–P–185720	California, Mexico Northwest	
Atriplex phyllostegia (Torr. ex S. Watson) S. Watson	UJ–KRA–P–40913–N / KRA–P–447968	Nevada	
Atriplex polycarpa (Torr.) S. Watson	1. UJ–KRA–P–40915–N / KRA–P–291241<br>2. UJ–KRA–P–40916–N / KRA–P–291240	Arizona, California, Mexico Northwest, Nevada, Utah	
Atriplex pusilla (Torr. ex S.Watson) S. Watson	UJ–KRA–P– 41388–N / KRA–P–360239	Nevada, Oregon<br><br>Extinct: California	
Atriplex elachophylla F. Muell.	1. UJ–KRA–P–40001–N / KRA–P–108706	New South Wales, Northern Territory,	

	2. UJ–KRA–P–40002–N / KRA–P–108707	Queensland, South Australia, Western Australia	
Atriplex quinii F. Muell.	UJ–KRA–P– 41389–N / KRA–P–109900	New South Wales, Northern Territory, Queensland, South Australia, Western Australia	
Bienertia cycloptera Bunge	1. UJ–KRA–P–42660–N / KRA–P–186277<br>2. UJ–KRA–P–42659–N / KRA–P–186278	Afghanistan, Iran, Kazakhstan, Pakistan, South European Russi, Transcaucasus, Turkey, Turkmenistan	
Celosia anthelminthica Asch.	UJ–KRA–P–35474–N / KRA–P–184385	Ethiopia, Kenya, Rwanda, Tanzania, Uganda, Yemen	
Celosia elegantissima Hauman	UJ–KRA–P–35478–N / KRA–P–110335	Burundi, Rwanda, Tanzania, Uganda, Zaïre	LC [9]
Celosia elongata Spreng.	UJ–KRA–P–35479–N / KRA–P–184393	Bolivia, Chile Central, Chile North, Colombia, Ecuador, Peru	
Chenopodium californicum (S. Watson) S. Watson	UJ–KRA–P–37952–N / KRA–P–440253	California, Mexico Northwest	
Chenopodium nitrariaceum (F.Muell.) F.Muell. ex Benth.	UJ–KRA–P–38624–N / KRA–P–113490	New South Wales, Northern Territory, Queensland, South Australia, Victoria	
Chenopodium sosnowskyi Kapeller	UJ–KRA–P–39364–N / KRA–P–185063	Iran, North Caucasus, Transcaucasus, Turkey, Turkmenistan	
Chenopodium desertorum (J. M. Black) J. M. Black	1. UJ–KRA–P–37968–N / KRA–P–112865<br>2. UJ–KRA–P–37969–N / KRA–P–112864<br>3. UJ–KRA–P–37970–N / KRA–P–112863	New South Wales, Northern Territory, Queensland, South Australia, Victoria, Western Australia	
Corispermum papillosum (Kuntze) Iljin	UJ–KRA–P–12636–N / KRA–P–470253	Kazakhstan, Tadzhikistan, Turkmenistan, Uzbekistan	
Cornulaca korshinskyi Litv.	UJ–KRA–P–42694–N / KRA–P–186291	Kazakhstan, Tadzhikistan, Turkmenistan, Uzbekistan	
Cyathula polycephala Baker	UJ–KRA–P–36716–N / KRA–P–184760	Ethiopia, Kenya, Rwanda, Tanzania, Zaïre	
Gamanthus commixtus Bunge	UJ–KRA–P–43122–N / KRA–P–186341	Afghanistan, Kazakhstan, Kirgizstan, Tadzhikistan, Uzbekistan	
Gamanthus ferganicus Iljin	1. UJ–KRA–P–12641–N / KRA–P–488421<br>2. UJ–KRA–P–12642–N / KRA–P–488413	Kirgizstan, Tadzhikistan, Uzbekistan<br><br>Endemic species of the Ferghana valley (Uzbekistan) [10]	
Gamanthus pilosus (Pall.) Bunge	UJ–KRA–P–43123–N / KRA–P–186069	North Caucasus, Transcaucasus	
Gomphrena nitida Rothr	1. UJ–KRA–P–36874–N / KRA–P–453883	Texas	

	2. UJ–KRA–P–36875–N / KRA–P–454004		
Gomphrena sonorae Torr.	UJ–KRA–P–36877–N / KRA–P–291245	Arizona, Mexico Northeast, Mexico Northwest, Mexico Southwest, New Mexico	
Gomphrena parviflora Benth.	UJ–KRA–P–36876–N / KRA–P–108662	Northern Territory, Western Australia	
Halanthium rarifolium K. Koch	1. UJ–KRA–P–43120–N / KRA–P–277995<br>2. UJ–KRA–P–43119–N / KRA–P–277316<br>3. UJ–KRA–P–43118–N / KRA–P–277315	Iran, North Caucasus, Transcaucasus, Turkey	
Halogeton sativus (L.) Moq	UJ–KRA–P–43127–N / KRA–P–186346	Algeria, Morocco, Spain	
Halosarcia pruinosa (Paulsen) Paul G. Wilson	UJ–KRA–P–43131–N / KRA–P–110753	Northern Territory, South Australia, Victoria, Western Australia	
Halothamnus hierochunticus (Bornm.) Botsch.	UJ–KRA–P–43132–N / KRA–P–129531	Iran, Iraq, Lebanon–Syria, Palestine, Turkey	
Kalidium gracile Fenzl	1. UJ–KRA–P–12651–N / KRA–P–108365<br>2. UJ–KRA–P–12650–N / KRA–P–100287<br>3. UJ–KRA–P–12652–N / KRA–P–107897	Inner Mongolia, Mongolia, Xinjiang	
Lagrezia monosperma (Rose) Standl.	1. UJ–KRA–P–35497–N / KRA–P–276227<br>2. UJ–KRA–P–35498–N / KRA–P–276229	Mexico Southeast, Mexico Southwest	LC [11]
Maireana aphylla (R. Br.) Paul G. Wilson	1. UJ–KRA–P–42339–N / KRA–P–108691<br>2. UJ–KRA–P–42338–N / KRA–P–108689	New South Wales, Northern Territory, Queensland, South Australia, Victoria, Western Australia<br><br>Endemic	
Maireana appressa Paul G. Wilson	1. UJ–KRA–P–42340–N / KRA–P–112900<br>2. UJ–KRA–P–42341–N / KRA–P–112899	New South Wales, Northern Territory, Queensland, South Australia, Victoria, Western Australia<br><br>Endemic	
Maireana georgei (Diels) Paul G. Wilson	UJ–KRA–P–42343–N / KRA–P–108687	New South Wales, Northern Territory, Queensland, South Australia, Victoria, Western Australia<br><br>Endemic	
Maireana integra (Paul G. Wilson) Paul G. Wilson	UJ–KRA–P–42342–N / KRA–P–108686	New South Wales, Northern Territory, South Australia, Western Australia	

		Endemic	
Maireana planifolia (F. Muell.) Paul G. Wilson	1. UJ–KRA–P–42345–N / KRA–P–108685<br>2. UJ–KRA–P–42344–N / KRA–P–108684	New South Wales, Northern Territory, South Australia, Western Australia<br><br>Endemic	
Maireana pyramidata (Benth.) Paul G. Wilson	UJ–KRA–P–42346–N / KRA–P–112870	New South Wales, South Australia, Victoria, Western Australia<br><br>Endemic	
Maireana sclerolaenoides (F. Muell.) Paul G. Wilson	UJ–KRA–P–42336–N / KRA–P–108683	New South Wales, Western Australia<br><br>Endemic	
Malacocera gracilis Chinnock	UJ–KRA–P–42069–N / KRA–P–113633	South Australia	
Ptilotus macrocephalus (R. Br.) Poir.	UJ–KRA–P–36749–N / KRA–P–109803	New South Wales, Northern Territory, Queensland, South Australia, Victoria, Western Australia	
Ptilotus obovatus (Gaudich.) F. Muell.	1. UJ–KRA–P–36751–N / KRA–P–112697<br>2. UJ–KRA–P–36754–N / KRA–P–113622<br>3. UJ–KRA–P–36752–N / KRA–P–113357<br>4. UJ–KRA–P–36753–N / KRA–P–113356	New South Wales, Northern Territory, Queensland, South Australia, Western Australia	
Rhagodia parabolica R. Br.	UJ–KRA–P–36920–N / KRA–P–112880	New South Wales, Northern Territory, Queensland, South Australia, Victoria, Western Australia	
Salicornia blackiana Ulbr.	UJ–KRA–P–43136–N / KRA–P–315636	South Australia, Tasmania, Victoria, Western Australia	
Salsola araneosa Botsch.	UJ–KRA–P–42714–N / KRA–P–186139	Namibia	
Salsola brevifolia Desf.	UJ–KRA–P–43017–N / KRA–P–186260	Algeria, Morocco, Tunisia	
Salsola capensis Botsch.	UJ–KRA–P–42728–N / KRA–P–417950	Cape Provinces	? [12]
*Salsola papillos*a (Coss.) Willk.	UJ–KRA–P–42933–N / KRA–P–186214	Spain	LC [13]
Salsola verticillata Schousb.	1. UJ–KRA–P–43034–N / KRA–P–253346<br>2. UJ–KRA–P–43033–N / KRA–P–405044<br>3. UJ–KRA–P–43030–N / KRA–P–353896<br>4. UJ–KRA–P–43031–N / KRA–P–353898<br>5. UJ–KRA–P–43032–N / KRA–P–353897	Morocco	

Salsola melitensis Botsch.	UJ–KRA–P–43047–N / KRA–P–460410	Sicilia<br><br>Endemic	
Sclerolaena johnsonii (Ising) A. J. Scott	1. UJ–KRA–P–42352–N / KRA–P–108711<br>2. UJ–KRA–P–42352–N / KRA–P–108711	New South Wales, Northern Territory, Queensland, South Australia, Western Australia	
Sclerolaena uniflora R. Br.	1. UJ–KRA–P–42355–N / KRA–P–108709<br>2. UJ–KRA–P–42354–N / KRA–P–110750	South Australia, Western Australia	
Seidlitzia rosmarinus Bunge ex Boiss.	UJ–KRA–P–42701–N / KRA–P–186292	Afghanistan, Egypt, Gulf States, Iran, Iraq, Kuwait, Lebanon–Syria, Oman, Palestine, Saudi Arabia, Sinai, Socotra, Tadzhikistan, Turkmenistan, Uzbekistan	
Suaeda californica S. Watson	UJ–KRA–P–42567–N / KRA–P–186121	California, Mexico Northwest	
Suaeda occidentalis (S. Watson) S. Watson	1. UJ–KRA–P–42676–N / KRA–P–312130<br>2. UJ–KRA–P–42677–N / KRA–P–312127	California, Idaho, Montana, Nevada, Oregon, Utah, Washington, Wyoming	
Traganum moquinii Webb ex Moq.	1. UJ–KRA–P–42691–N / KRA–P–417495<br>2. UJ–KRA–P–42689–N / KRA–P–426192<br>3. UJ–KRA–P–42690–N / KRA–P–426494	Canary Is., Cape Verde, Mauritania, Morocco, Western Sahara	

References:

1. POWO. Plants of the World Online, Facilitated by the Royal Botanic Gardens, Kew. Available online: ttp://www.plantsoftheworldonline.org/ (accessed on 10 October 2023).
2. Thiv, M.; Thulin, M.; Kilian, N.; Linder, H.P. Eritreo–Arabian Affinities of the Socotran flora as revealed from the moolecular phylogeny of *Aerva* (*Amaranthaceae*). Syst. Bot. **2006**, *31*(3), 560–570. https://www.jstor.org/stable/25064185.
3. Miller, A. *Aerva microphylla*. The IUCN Red List of Threatened Species 2004: e.T44753A10946247. Available online: https://www.iucnredlist.org/species/44753/10946247 (accessed on 10 October 2023).
4. Weakley, A.; Bucher, M.; Murdock, N. Recovery Plan for Seabeach Amaranth (*Amaranthus pumilus*); U.S. Fish and Wildlife Service, Southeast Region: Atlanta, GA, 1996; pp 1–59.
5. Marcone, M. F. First report of the characterization of the threatened plant species *Amaranthus pumilus* (seabeach amaranth). *J. Agric. Food Chem.* **2000**, *48*(2), 378–382. DOI: 10.1021/jf991133a.
6. NSE. Nature Serve Explorer. Available online: https://explorer.natureserve.org/Taxon/ELEMENT_GLOBAL.2.141860/Amaranthus_pumilus (accessed on 10 Mach 2024).
7. IUNC. The IUCN Red List of Threatened Species 2004. Available online: https://www.iucnredlist.org/species/199895/2617800 (accessed on 07 March 2024).
8. Hadjiev, V. *Anabasis eugeniae*. The IUCN Red List of Threatened Species 2014: Available online: https://www.iucnredlist.org/species/199895/2617800 (accessed on 07 March 2024).

9. Ntore, S.; Beentje, H.J.; Fischer, E.; Kabuye, C.; Kalema, J.; Kayombo, C.; Luke, W.R.Q.; Nshutiyayesu, S. *Celosia elegantissima*. The IUCN Red List of Threatened Species 2019: Available online: https://www.iucnredlist.org/species/103581524/103648247 (accessed on 07 March 2024).
10. Foziljonov S. Theoretical analysis of the degree of scarity of representatives of the family of *Chenopodiaceae* in the Feragana Valley. *Cent. Asian J. Med. Sci.* **2021**, *2*(3), 295–299.
11. Martínez Salas, E.; Fuentes, A.C.D.; Samain, M.–S. *Celosia monosperma*. The IUCN Red List of Threatened Species 2020: Available online: https://www.iucnredlist.org/species/136621713/137375989.
12. Raimondo, D.; von Staden, L.; Foden, W.; Victor, J.E.; Helme, N.A.; Turner, R.C.; Kamundi, D.A.; Manyama, P.A. Red List of South African Plants. Strelitzia 25. South African National Biodiversity Institute, Pretoria, 2009.
13. Buira, A.; Fraga i Arquimbau, P. *Salsola papillosa*. The IUCN Red List of Threatened Species 2017: Available online: https://www.iucnredlist.org/species/103535407/103535411 (accessed on 10 October 2022).
