# Supplementary material for: Geographical–Historical Analysis of the Herbarium Specimens Representing the Economically Important Family Amaranthaceae (Chenopodiaceae-Amaranthaceae Clade) Collected in 1821–2022 and Preserved in the Herbarium of the Jagiellonian University in Krakow
Source: Biology (Basel). 2024 Jun 13;13(6):435. doi: 10.3390/biology13060435 (PMC11201225; doi:10.3390/biology13060435)
Supplement: Supplementary file 1 [file biology-13-00435-s001.zip › Table S3.pdf]

Table S3. List of economically useful species based on herbarium sheets of the *Amaranthaceae* family deposited in the KRA herbarium.

Name of species	Native to [1]:	Uses	Parts of the plant	Selected references
Achyrantches aspera L.	Africa, south Asia and Australia, same country in Europe	Medicinal properties	all parts of the plant	[2–8]
Aerva javanica (Burm.f.) Juss. ex Schult.	Afica, south–west Asia	Medicinal properties	all parts of the plant	[9,10]
		Reclamation and regeneration of grasslands in areas degraded by grazing		[11]
		For stuffing pillows and scoops	flowers	[12]
Aerva lanata (L.) Juss.	Africa, south Asia	Medicinal properties	all parts of the plant	[5,13,14]
Aerva leucura Moq.	South Africa	For stuffing pillows and scoops	flowers	[12]
Agriophyllum squarrosum (L.) Moq.	East European Russia, Irkutsk, Kazakhstan, Krasnoyarsk, South European Russi, West Siberia	Food	seeds	[15]
		Animal feed	aerial parts of the plant	[15]
		Medicinal properties	seeds	[16–18]
		As a pioneer plant on dunes in arid or semi–arid regions		[16–18]
Allmania nodiflora (L.) R.Br. ex Wight	Assam, Borneo, China Southeast, East Himalaya, Hainan, India, Jawa, Laos, Lesser Sunda Is., Malaya, Philippines, Sri Lanka, Sumatera, Thailand, Vietnam	Food	leaves	[19]
		Medicinal properties		[19,20]
Alternanthera caracasana Kunth	Middle and South America	Medicinal properties	all parts of the plant	[21]
		Potential source of bioethanol for fuel production	all parts of the plant	[22]

Alternanthera philoxeroides (Mart.) Griseb.	North and Central South America	Medicinal properties		[13]
Alternanthera pungens Kunth	Middle and South America	Potential medicinal properties	aerial parts of the plant	[23]
Alternanthera sessilis (L.) R.Br. ex DC.	Middle and South America	Food	weed	[23]
		Medicinal properties	weed, leaves	[13,24–26]
Alternanthera porrigens (Jacq.) Kuntze	Bolivia, Chile Central, Chile North, Colombia, Ecuador, Peru	Medicinal properties	leaves, flowers	[27]
Amaranthus blitum L.	Argentina Northeast, Argentina Northwest, Bolivia, Brazil Northeast, Brazil South, Brazil Southeast, Brazil West–Central, Chile North, Paraguay, Peru, Uruguay	Food	leaves	[28–30]
		Medicinal properties		[13]
Amaranthus caudatus L.	Argentina Northwest, Bolivia, Ecuador, Peru	Food	seeds (pseudożboże)	[28,29]
Amaranthus cruentus L.	El Salvador, Guatemala, Honduras, Mexico Central, Mexico Gulf, Mexico Southeast, Mexico Southwest, Nicaragua	Medicinal properties	stems, leaves,	[1,27,31]
		Food	seeds (pseudożboże)	[1,28,29]
		Animal feed		[1]
Amaranthus dubius Mart. ex Thell.	Mexico to Tropical South America	Food	leaves	[28,29]
Amaranthus hybridus L.	S. Ontario to W. South America	Medicinal properties	leaves, flowers	[27]
Amaranthus hypochondriacus L.	Kansas, Mexico Central, Mexico Gulf, Mexico Northeast, Mexico Northwest, Mexico Southwest, Minnesota, Missouri, Nebraska, Texas, Wisconsin	Food	seeds (pseudożboże)	[28,29]
Amaranthus retroflexus L.	Mexico Central, Mexico Gulf, Mexico Northeast, Mexico Southeast, Mexico Southwest	Food	leaves, fruit	[32]
Amaranthus spinosus L.	Mexico to Tropical South America	Medicinal properties	all parts of the plant	[13,31,33]

		Food	leaves	[28,29]
Amaranthus tricolor L.	Assam, Bangladesh, Borneo, India, Jawa, Laos, Lesser Sunda Is., Line Is., Malaya, Maluku, Myanmar, Nepal, New Guinea, Philippines, Solomon Is., Sri Lanka, Sulawesi, Sumatera, Thailand, Vietnam	Medicinal properties		[13]
		Food	leaves	[28,29]
Amaranthus viridis L.	SE. Mexico to Tropical South America	Food	leaves	[28,29]
		Medicinal properties	roots, leaves, seeds	[5,13,14]
Anabasis aphylla L.	China North–Central, Iran, Kazakhstan, Kirgizstan, North Caucasus, South European Russi, Tadzhikistan, Transcaucasus, Turkmenistan, Uzbekistan, Xinjiang	Medicinal properties	stems	[34,35]
Anabasis articulata (Forssk.) Moq.	Algeria, Egypt, Gulf States, Iraq, Lebanon–Syria, Libya, Mauritania, Morocco, Palestine, Saudi Arabia, Sinai, Spain, Tunisia, Western Sahara	Medicinal properties		[36]
		As fuel (woody specimens)		
Anabasis salsa (Ledeb.) Benth. ex Volkens	Altay, East European Russia, Iran, Kazakhstan, Mongolia, South European Russi, Tadzhikistan, Transcaucasus, Turkmenistan, Uzbekistan, Xinjiang	Production of insecticides		[37]
Arthrocnemum macrostachyum (Moric.) K.Koch	Macaronesia, Medit. to S. Pakistan and Senegal, Angola, Arabian Peninsula	Medicinal properties	leaves, seeds	[38]
		Potential use in phytoremediation of cadmium–contaminated soils		
Atriplex atacamensis Phil.	Chile North, Peru	Potential use for phytoremediation of arsenic–contaminated sites		[39]

Atriplex cana Ledeb.	Altay, East European Russia, Kazakhstan, Kirgizstan, Krym, North Caucasus, South European Russi, Tadzhikistan, Transcaucasus, West Siberia, Xinjiang	Animal feed		[40]
Atriplex canescens (Pursh) Nutt.	W. Central Canada to Mexico : Alberta, Arizona, California, Colorado, Idaho, Kansas, Mexico Central, Mexico Gulf, Mexico Northeast, Mexico Northwest, Mexico Southeast, Montana, Nebraska, Nevada, New Mexico, North Dakota, Oklahoma, Oregon, South Dakota, Texas, Utah, Washington, Wyoming	Soil reclamation		[41]
Atriplex farinosa Forssk.	Egypt to NE. Tropical Africa, Arabian Peninsula: Djibouti, Egypt, Eritrea, Gulf States, Kenya, Oman, Saudi Arabia, Sinai, Socotra, Somalia, Sudan, Yemen	Medicinal properties		[42]
		Tested for insecticide production vs *Helicoverpa zea*		[43]
Atriplex glauca L.	Algeria, Canary Is., Egypt, Lebanon–Syria, Libya, Madeira, Mauritania, Morocco, Palestine, Portugal, Saudi Arabia, Sinai, Spain, Tunisia, Western Sahara	Medicinal properties	leaves	[43]
		Food	leaves (also dried)	[44]
		Animal feed		[41]
Atriplex gmelinii C.A.Mey. ex Bong.	Alaska, British Columbia, California, Kamchatka, Khabarovsk, Kuril Is., Magadan, Northwest Territorie, Oregon, Washington	Potential medicinal properties		[45]
Atriplex halimus L.	Algeria, Baleares, Canary Is., Cape Verde, Corse, Cyprus, East Aegean Is., Egypt, Eritrea, Ethiopia, France, Greece, Iraq, Italy, Kriti, Lebanon–	Phytoextraction of heavy metals (arsenic–contaminated areas)		[39,46,47]
		Combating internal parasites (veterinary)		[43]

	Syria, Libya, Madeira, Mali, Mauritania, Morocco, Palestine, Portugal, Sardegna, Saudi Arabia, Sicilia, Sinai, Somalia, Spain, Tunisia, Turkey, Western Sahara	Medicinal properties		[38,48]
Atriplex hortensis L.	East European Russia, Iran, Kazakhstan, North Caucasus, South European Russi, Transcaucasus	Medicinal properties	leaves	[49]
		Food	leaves	[44]
Atriplex leucoclada Boiss.	Afghanistan, Egypt, Gulf States, Iran, Iraq, Kazakhstan, Kuwait, Lebanon–Syria, Oman, Pakistan, Palestine, Saudi Arabia, Sinai, Transcaucasus, Turkey, Turkmenistan, Uzbekistan, Yemen	Medicinal properties	leaves	[50]
Atriplex lindleyi Moq.	Australia	Medicinal properties		[51,52]
Atriplex prostrata Boucher ex DC.	Macaronesia to N. Xinjiang, E. Canada to E. U.S.A	Food	leaves	[32]
		Medicinal properties		[38]
Atriplex rosea L.	Europe to Medit. and Central Asia	Food	leaves	[32]
Atriplex sagittata Borkh.	Central and E. Europe to Central Asia	Food	leaves	[32]
Atriplex semibaccata R.Br.	Australia	improving vineyard productivity (agroforestry)<br>groundcover for places with a lack of water		[32]
Bassia muricata (L.) Asch.	E. Medit., N. and NE. Africa, Arabian Peninsula	Medicinal properties	stems, flowers, seeds	[36,53]
Beta vulgaris L.	Azores, W. Europe to Medit. and India			
Chenopodium acerifolium Andrz.	Poland to Siberia	Medicinal properties	aerial parts of plant	[38]
Chenopodium album L.	Temp. to Indian Subcontinent	Medicinal properties	aerial part of plant	[5,14, 27]
		Food	leaves, fruits	[31,32]

Chenopodium bonus–henricus L.	Europa	Food	roots, leaves, flowers	[32,54]
		Medicinal properties	roots	[54]
Chenopodium polyspermum L.	Europe, E. Medit. to Siberia	Food	leaves	[32]
Chenopodium quinoa Willd.	Argentina Northwest, Bolivia, Chile North, Ecuador, Peru	Food	leaves, seeds (pseudozboże)	[55–59]
Chenopodium urbicum L.	Europe to Russian Far East and N. China	Food	leaves	[32]
Cornulaca monacantha Delile	Sahara to W. and SW. Pakistan	Medicinal properties	twigs, leaves	[36,53]
Dysphania aristata (L.) Mosyakin & Clemants	European Russia to Korea	Rapid growth generates a large amount of biomass, which is important when using the plant to synthesize AgNP silver nanoparticles (antibacterial effect)	stems	[60]
Gomphrena globosa L.	Brazil, Panama and Guatemala	Medicinal properties	leaves, flowers	[13,61–63]
Gomphrena celosioides Mart.	South America (Ecuador to N. Argentina)	Medicinal properties		[13]
Halocnemum strobilaceum (Pall.) M.Bieb.	N. Central Italy to SW. Siberia and N. China, NE. Tropical Africa to Arabian Peninsula	Medicinal properties	aerial parts of the plant	[36]
Halosarcia indica (Willd.) Paul G.Wilson	Coasts of Dry Tropical Africa to KwaZulu–Natal, Madagascar, Indian Subcontinent, Vietnam, Jawa to N. Australia	Medicinal properties		[5]
Halostachys belangeriana (Moq.) Botsch.	E. Türkiye to N. China and SW. Pakistan	Medicinal properties	leaves	[34]
Haloxylon ammodendron (C.A.Mey.) Bunge ex Fenzl	Afghanistan, China North–Central, Inner Mongolia, Iran, Kazakhstan, Kirgizstan, Mongolia, Qinghai, Tadzhikistan, Turkmenistan, Uzbekistan, Xinjiang	Medicinal properties	all plants	[34]
		Afforestation of dry areas in China and planted as shelter belts and for consolidating dunes		[34]
		Storage of water used during drought		[34]
Iresine herbstii Hook.	Peru	Medicinal properties	flowers	[27]

Noaea mucronata (Forssk.) Asch. & Schweinf.	Afghanistan, Algeria, Cyprus, East Aegean Is., Egypt, Greece, Iran, Iraq, Kriti, Lebanon–Syria, Libya, Morocco, North Caucasus, Palestine, Saudi Arabia, Sinai, Transcaucasus, Turkey, Turkey–in–Europe, Turkmenistan, Uzbekistan	Medicinal properties	leaves, flowers	[50]
Salsola imbricata Forssk.	Afghanistan, Algeria, Chad, Djibouti, Egypt, Eritrea, Gulf States, India, Iran, Iraq, Kuwait, Lebanon–Syria, Libya, Mauritania, Morocco, Niger, Oman, Pakistan, Palestine, Saudi Arabia, Senegal, Sinai, Somalia, Sudan, Western Sahara, Yemen	Medicinal properties		[5]
Salsola vermiculata L.	Algeria, Baleares, Canary Is., Djibouti, Iran, Iraq, Lebanon–Syria, Mauritania, Morocco, Niger, Palestine, Portugal, Sardegna, Sicilia, Spain, Sudan, Transcaucasus, Tunisia<br><br>southwest of Asia	Medicinal properties	stem, leaves, roots, seeds	[50,64]
Sarcocornia fruticosa (L.) A.J.Scott	Albania, Algeria, Baleares, Canary Is., Corse, Cyprus, Egypt, France, Greece, Iran, Italy, Lebanon–Syria, Libya, Morocco, Palestine, Portugal, Sardegna, Sicilia, Sinai, Spain, Tunisia, Turkey, Turkey–in–Europe, Yemen, Yugoslavia	Medicinal properties	all plants	[38]
Spinacia oleracea L.	Afghanistan, Iran, Kazakhstan, Kirgizstan, Pakistan, Tadzhikistan, Turkmenistan, Uzbekistan	Food	leaves	[65]

Suaeda vermiculata Forssk. ex J.F.Gmel.	Afghanistan, Algeria, Canary Is., Chad, Djibouti, Egypt, Eritrea, Gulf States, India, Iran, Iraq, Kuwait, Libya, Mauritania, Morocco, Oman, Palestine, Saudi Arabia, Senegal, Sicilia, Sinai, Socotra, Somalia, Spain, Sri Lanka, St.Helena, Sudan, Tunisia, Western Sahara, Yemen	Medicinal properties	all plants	[5,38]
Suaeda maritima (L.) Dumort.	Central & E. Canada to E. U.S.A., Europe to W. Siberia, Macaronesia, N. Africa to Indo–China	Medicinal properties	aerial parts of the plant	[38]
Suaeda monoica Forssk. ex J.F.Gmel.	Afghanistan, Botswana, Cape Provinces, Chad, Djibouti, Egypt, Eritrea, Ethiopia, Free State, India, Iran, Iraq, Kenya, Lebanon–Syria, Mozambique, Namibia, Oman, Pakistan, Palestine, Saudi Arabia, Sinai, Socotra, Somalia, Sri Lanka, Sudan, Tanzania, Yemen	Medicinal properties	aerial parts of the plant	[5]
Traganum nudatum Delile	Algeria, Egypt, Gulf States, Iraq, Kuwait, Lebanon–Syria, Libya, Mauritania, Morocco, Palestine, Saudi Arabia, Sinai, Tunisia, Western Sahara	Medicinal properties	stems, leaves	[36,53]
		Food	fruits	[36]
		Fuel	all plants	[36]

References:

1. POWO. Plants of the World Online, Facilitated by the Royal Botanic Gardens, Kew. Available online: http://www.plantsoftheworldonline.org/ (accessed on 10 January 2024).
2. Shafique, S; Javaid, A; Bajwa, R.; Shafiqe, S. Biological control of *Achyranthes aspera* and *Xanthium strumarium* in Pakistan. *Pak. J. Bot.* **2007**, *39*(7), 2607–2610.
3. Sumeet, D.; Raghvendra, D.; Kushagra, M. *Achyranthes aspera* Linn. (Chirchira): A magic herb in folk medicine. *Ethnobotanical Leaflets* **2008**, *12*, 670–676. DOI: 10.7439/ijbar.v2i6.36.
4. Varuna, K.M.; Khan, M.U.; Sharma, P.K. Review on *Achyranthes aspera*. *J. Pharm. Pract. Res*. **2010**, *3*(4), 714–171.

5. Ozturk, M.; Mermut, A. R.; Celik, A. Urbanisation, land use, land degradation and environment. *Ambio* **2011**, *51*, 1446–1458. DOI: 10.1007/s13280–022–01701–7.
6. Mukherjeea, H.; Ojhaa, D.; Baga, P.; Chandelb, H.S.; Bhattacharyyad, S.; Chatterjeed, T.K.; Mukherjeed, P.K.; Chakrabortia, S.; Chattopadhyaya, D. Anti–herpes virus activities of *Achyranthes aspera*: An Indian ethnomedicine, and its triterpene acid. *Microbiol. Res.* **2013**, *168*, 238–244. DOI: 10.1016/j.micres.2012.11.002.
7. Hasan S. Pharmacological and medicinal uses of *Achyranthes aspera*. *Int. J. Sci. Environ*. **2014**, *3*, 123–129.
8. Singh, N.; Mrinal, Sharma P.; Gupta, V.K. A review on pharmacological aspects of *Achyranthes aspera*. *International Journal of Pharmacognosy and Chinese Medicine* **2019**, *3*(4), 000188. DOI: 10.23880/ipcm–16000188.
9. Movaliya, V.; Zaveri, M. A review on the pashanbheda plant "*Aerva javanica*." *Int. J. Pharm. Sci. Rev. Res*. **2014**, *25*(2), 268–275.
10. Suleiman, M.H.A. Ethnobotanical, phytochemical and biological study of *Tamarix aphylla* and *Aerva javanica* medicinal plants growing in the Asir Region, Saudi Arabia. Trop. *Conserv. Sci.* **2019**, *12*, 1–14. DOI:10.1177/1940082919869480.
11. Novelly, P.; Watson, I. Successful grassland regeneration in a severely degraded catchment: a whole of government approach in north west Australia. Chapter 26 in: Climate and Land Degradation. Environmental science and engineering series. (Eds: M. V. K. Sivakumar and N. Ndiang'ui). Springer, Berlin, 2007, pp 469– 484.
12. Soliman, M.A. Cytogenetical studies on *Aerva javanica* (*Amaranthaceae*). *Fl. Medit.* **2006**, *16*, 333–339.
13. Rahman, A.H.M.M.; Gulshana, M.I.A. Taxonomy and medicinal uses on *Amaranthaceae* family of Rajshahi, Bangladesh. *Appl. Ecol. Environ. Res*. **2014**, *2*(2), 54–59. DOI: 10.12691/aees–2–2–3.
14. Qureshi, R.; Raza Bhatti, G. Ethnobotany of plants used by the Thari people of Nara Desert, Pakistan. *Fitoterapia* **2008**, *79*(6), 468–473. DOI: 10.1016/j.fitote.2008.03.010.
15. Zhao, P; Li, X.; Sun, H.; Zhao, X.; Wang, X.; Ran, R.; Zhao, J.; Wei, Y.; Liu, X.; Chen, G. Healthy values and de novo domestication of sand rice (*Agriophyllum squarrosum*), a comparative view against *Chenopodium quinoa*. *Crit. Rev. Food. Sci. Nutr*. **2023**, *63*(19), 4188–4209. DOI: 10.1080/10408398.2021.1999202.
16. Qian, C.; Yin, H.; Shi, Y.; Zhao, J.; Yin, C.; Luo, W.; Dong, Z.; Xia Yan, G.C.; Wang, X.–R.; Ma X.–F. Population dynamics of *Agriophyllum squarrosum*, a pioneer annual plant endemic to mobile sand dunes, in response to global climate change. *Sci. Rep*. **2016**, *6*, 26613. DOI: 10.1038/srep26613.
17. Genievskaya, Y.; Abugalieva, S.; Zhubanysheva, A.; Turuspekov, Y. Morphological description and DNA barcoding study of sand rice (*Agriophyllum squarrosum, Chenopodiaceae*) collected in Kazakhstan. *BMC Plant Biol.* **2017**, *17*(S1), 177. DOI: 10.1186/s12870–017– 1132–1.
18. Yin, X.; Yan, X.; Qian, C.; Zhou, S.; Fang, T.; Fan, X.; Gao, Y.; Chang, Y.; Yang, J.; Ma, X.F. Comparative transcriptome analysis to identify genes involved in terpenoid biosynthesis in *Agriophyllum squarrosum*, a folk medicinal herb native to Asian temperature deserts. *Plant Biotechnol. Rep.* **2021**, *15*(3), 369–387. DOI: 10.1007/s11816–021–00674–5.
19. Dhanya, V.; Ragavendran, U.; Aathira, M.; Saipriya, V.; Priya, M.S.; Palanisamy, S.; Siddhraju, P. Biochemical composition and antioxidant potential of raw and hydrothermal treated of two underutilized leafy vegetables *Amaranthus dubius* and *Allmania nodiflora*. *Int. J. Food Sci. Nut*. **2017**, *2*(4), 95–107.
20. Banerjee, S.; Joglekar, A.; Mishra, M. A critical review on importance of green leafy vegetables. *Int. J. Appl. Sci.* **2015**, *2*(3–4), 124–132. http://scientificresearchjournal.com/wp–content/uploads/2015/09/Home–Science–Vol–2_A–124–132–Full–Paper.pdf.

21. Canales–Martínez, M.; Hernández–Delgado, T.; Flores–Ortiz, C.; Durán–Díaz, A.; García–Bores, A.M.; Avila–Acevedo, G. Antimicrobial activity of *Alternanthera caracasana*. *Pharm. Biol.* **2005**, *43*(4), 305–307. DOI: 10.1080/13880200590951685.
22. Malthesh, S.R.Y.; Achar, R.R.; Cathrine, A.A.; Vadiraj, K.T. Extraction of alcohols from non–edible agricultural weed, lignocellulouic feedstock – *Alternanthera caracasana*. *World J. Environ. Bioscien*. **2023**, *12*(3), 27–32. DOI: 10.51847/OEAOcF6m7y.
23. Hwong, C. S.; Leong, K. H.; Abdul Aziz, A.; Mat Junit, S.; Mohd Noor, S.; Kong, K.W. *Alternanthera sessilis*: Uncovering the nutritional and medicinal values of an edible weed. *J. Ethnopharmacol*. **2022**, *298*, 115608. DOI: 10.1016/J.JEP.2022.115608.
24. George, S.; Bhalerao, S.V.; Lidstone, E.A.; Ahmad, I.S.; Abbasi, A.; Cunningham, B.T.; Watkin, K.L. Cytotoxicity screening of Bangladeshi medicinal plant extracts on pancreatic cancer cells. *BMC Complement Altern Med*. **2010**, *17*, 10–52. DOI: 10.1186/1472–6882–10–52.
25. Hossain, A.I.; Faisal, M.; Rahman, S.; Jahan, R.; Rahmatullah, M. A preliminary evaluation of antihyperglycemic and analgesic activity of *Alternanthera sessilis* aerial parts. *BMC Complement Altern Med*. **2014**, *24*, 14–169. DOI: 10.1186/1472–6882–14–169.
26. Thomas, W.; Merish, S.; Tamizhamuthu, M. Review of Alternanthera sessilis with reference to traditional siddha medicine. *Int. J. Pharmacogn. Pharm. Res.* **2014**, *6*(2), 249–254.
27. Tene, V.; Malagón, O.; Finzi, P.V.; Vidari, G.; Armijos, C.; Zaragoza, T. An ethnobotanical survey of medicinal plants used in Loja and Zamora–Chinchipe, Ecuador. *J. Ethnopharmacol*. **2007**, *111*(1), 63–81. DOI: 10.1016/j.jep.2006.10.032.
28. Mosyakin, S.; Robertson K. Amaranthus L. – In: Flora of North America Editorial Committee (ed.), Flora of North of Mexico (*Magnoliophyta*: *Caryophyllidae*, part 1). Oxford Universuty Press, Oxford, 2003, pp. 410–435.
29. Aderibigbe, O.R.; Ezekiel O.O.; Owolade, S.O.; Korese, J.K.; Sturm, B.; Hensel, O. Exploring the potentials of underutilized grain amaranth (*Amaranthus* spp.) along the value chain for food and nutrition security: A review. *Crit. Rev. Food Sci. Nutr.* **2020**, *62*(3), 656–669. DOI: 10.1080/10408398.2020.1825323.
30. Sarker, U.; Oba, S. Nutrients, minerals, pigments, phytochemicals, and radical scavenging activity in *Amaranthus blitum* leafy vegetables. *Sci. Rep*. **2020**, *10*(1), 1–9. DOI: 10.1038/s41598–020–59848–w.
31. Abbasi, A.; Khan, M.; Shah, M.H.; Shah, M.; Pervez, A.; Ahmad, M. Ethnobotanical appraisal and cultural values of medicinally important wild edible vegetables of Lesser Himalayas–Pakistan. *J. Ethnobiol. Ethnomed*. **2013**, *9*(1), 66. DOI: 10.1186/1746–4269–9–66.
32. Nedelcheva, A. An ethnobotanical study on wild edible plants in Bulgaria. *Eurasian J. Biosci.* **2013**, *7*, 77–94. DOI: 10.5053/ejobios.2013.7.0.10.
33. Ganjare, A.B.; Raut, N.A. Nutritional and medicinal potential of *Amaranthus spinosus*. *J. Pharmacogn. Phytochem*. **2019**, *8*(3), 3149–3156. https://www.phytojournal.com/archives/2019/vol8issue3/PartAS/8–3–128–378.pdf.
34. Abdiniyazova G.J.; Khojimatov O.K. Medicinal plants of Karakalpakstan. *Int. J. Sci. Res*. **2017**, *6*(11), 2113–2116. DOI: 10.21275/art20178256.
35. Shakeri, A.; Hazeri, N.; Vlizadeh, J.; Ghasemi, A.; Tavallaei, F.Z. Phytochemical screening, antimicrobial and antioxidant activities of *Anabasis aphylla* extracts. *Kragujevac J. Sci.* **2012**, *34*(34), 71–78.
36. Lakhdari, W.; Dehliz, A.; Acheuk, F.; M'lik, R.; Hammi, H.; Doumandji–mitiche, B.; Gheriani, S.; Berrekbia, M.; Guermit, K.; Chergui, S. Ethnobotanical study of some plants used in traditional medicine in the region of Oued Righ (Algerian Sahara). *J. Med. Plants Stud*. **2016**, *4*, 204–211.
37. Pei, Y.; Yang, Z.D.; Sheng, J. Chemical constituents of *Anabasis salsa*. *Chem. Nat. Compd*. **2014**, *50*(5), 957–958. DOI: 10.1007/s10600–014–1132–4.
38. Megharbi, A.; Kechairi, R. Ethnobotanical characterization of halophytes with medicinal virtues, Case of the Macta wetland flora: North–West Algeria. *Genet. Biodivers. J.* **2021**, *5*(2), 135–145. DOI: 10.46325/gabj.v5i2.82.

39. Vromman, D.; Flores–Bavestrello, A.; Šlejkovec, Z.; Lapaille, S.; Teixeira–Cardoso, C.; Briceño, M.; Lutts, S. Arsenic accumulation and distribution in relation to young seedling growth in *Atriplex atacamensis* Phil. *Sci. Total Environ.* **2011**, *412–413*, 286–295. DOI:10.1016/j.scitotenv.2011.09.085.
40. DanHong C.; Cheng Y.; Yan W. Seed germination characteristics of the desert subshrub *Atriplex cana* and its ecological significance. *Acta Petrol. Sin.* **2015**, *24*(10), 131–138.
41. Ma, D.; He, Z.; Bai, X.; Wang, W.; Zhao, P.; Lin, P.; Zhou, H. *Atriplex canescens*, a valuable plant in soil rehabilitation and forage production. A review. *Sci. Total. Environ.* **2022**, *15*(804), 150287. DOI: 10.1016/j.scitotenv.2021.150287.
42. Soliman, G.A.; Abd El Raheim, M. Antihyperglycemic, antihyperlipidemic and antioxidant effect of *Atriplex farinosa* and *Atriplex nummularia* in streptozotocin–induced diabetes in rats. *Bull. Env. Pharmacol. Life Sci.* **2015**, *4*, 10–18.
43. Donia, A. E. R. M.; Alqasoumi, S. I.; Radwan, A. M.; Bur, J.; Craker, L. E. Phytochemical screening and insecti–cidal activity of three plants from *Chenopodiaceae* family. *J. Med. Plants Res*. **2012**, *6*(48), 5863–5867.
44. Karous, O.; Ben Haj Jilani, I.; Ghrabi–Gammar, Z. Ethnobotanical study on plant used by Semi–Nomad Descendants' Community in Ouled Dabbeb – Southern Tunisia. *Plants* **2021**, *10*, 642. DOI: 10.3390/plants10040642.
45. Jeong, H.; Kim, H.; Ju, E.; Kong, C.–S.; Seo, Y. Antioxidant effect of the halophyte *Atriplex gmelinii*. *KSBB J.* **2016**, *31*(4), 200–207. DOI: 10.7841/ksbbj.2016.31.4.200.
46. Lefèvre, I.; Marchal, G.; Meerts, P.; Corréal, E.; Lutts, S. Chloride salinity reduces cadmium accumulation by the Mediterraneanhalophyte species *Atriplex halimus* L. *Environ. Exp. Bot.* **2009**, *65*, 142–152. DOI: 10.1016/j.envexpbot.2008.07.005.
47. Manousaki, E.; Kalogerakis, N. Phytoextraction of Pb and Cd by the Mediterranean saltbush (*Atriplex halimus* L.): metal uptake in relation to salinity. *Environ. Sci. Pollut. Res. Int*. **2009**, *16*(7), 844–54. DOI: 10.1007/s11356–009–0224–3.
48. Soumia, H.; Zahia, B.; Aminata, O.E.H.K.; Hanane, B.; Zohra, B.; Yasmina, B.; Insaf, A.K.; Sara, M.; Fatma, S.; Meriem, T. Ethnobotanical study and phytochemical screening of six medicinal plants used in traditional medicine in the Northeastern Sahara of Algeria (area of Ouargla). *J. Med. Plant Res.* **2015**, *9*(41), 1049–1059. DOI: 10.5897/jmpr2015.5932.
49. Mardi, P.; Fallah Huseini, H.; Ahvazi, M.; Tavakoli–Far, B. Effects of *Atriplex hortensis* hydroalcoholic extract on phenyl–hydrazine induced hemolytic anemia in rat. *J. Med. Plants and By–product* **2022**, *11*, 37–41. DOI: 10.22092/jmpb.2020.352222.1276.
50. Ghasemi, P.; Momeni, M.; Bahmani, M. Ethnobotanical study of medicinal plants used by Kurd tribe in Dehloran and Abdanan districts, Ilam province, Iran. *Afr. J. Tradit. Complement Altern. Med.* **2013**, *10*(2). DOI: 10.4314/ajtcam.v10i2.24.
51. El Souda, S.S.; Matloub, A.A.; Nepveu, F.; Valentin, A.; Roques, C. Phenolic composition and prospective anti–infectious properties of *Atriplex lindleyi*. *Asian Pac. J. Trop. Dis.* **2015**, *5*, 786–791. DOI: 10.1016/S2222–1808(15)60931–8.
52. Matloub, A. A.; Hamed, M. A.; El Souda, S.S. Chemo–protective effect on hepato–renal toxcicity and cytotoxic activity of lipoidal matter of *Atriplex lindleyi* Moq. *Int. J. Pharm. Pharm. Sci.* **2014**, 6(8), 187–196.
53. Alharbi, N. Survey of plant species of medical importance to treat digestive tract diseases in Tabuk Region, Saudi Arabia. *J. King Abdulaziz Univ. Sci.* **2017**, *29*(1), 51–61. DOI: 10.4197/sci.29–1.6.
54. Hanganu, D.; Olah, N.; Dubei, N.; Mărculescu, A. Phytochemical studies on *Chenopodium bonus–henricus* L. *Proc. Rom. Acad., Series B,* **2008**, *3*, 287–292.
55. Mabberley, D.J. The plant book. Cambridge: Cambridge University Press, 1993, pp. 119.

56. Koziok, M.J. Chemical composition and nutritional evaluation of quinoa (*Chenopodium quinoa* Willd.). *Food Comp. Anal.* **1992**, *5*, 35–68.
57. Schlick, G.; Bubenheim, D. L. Quinoa: Candidate crop for NASA's Controlled Ecological Life Support Systems. In Progress in New Crops; Janick, J., Ed.; ASHS Press: Arlington, VA, 1996.
58. Dini, I.; Schettino, O.; Simioli, T.; Dini, A. Studies on the constituents of *Chenopodium quinoa* seeds: Isolation and characterization of new triterpene saponins. *J. Agric. Food Chem*. **2001**, *49*(2), 741–746. DOI: 10.1021/jf000971y.
59. Iqbal, M. An assessment of quinoa (*Chenopodium quinoa* Willd.) potential as a grain crop on marginal lands in Pakistan. *Am. Eurasian J. Agric. Environ. Scies.* **2015**, *15*(1): 16–23. DOI: 10.5829/idosi.aejaes.2015.15.1.12474.
60. Yuan, C.–G.; Huo, C.; Gui, B.; Liu, P.; Zhang, C. Green synthesis of silver nanoparticles using *Chenopodium aristatum* L. stem extract and their catalytic/antibacterial activities. *J. Cluster Sci.* **2016**, *28*(3), 1319–1333. DOI: 10.1007/s10876–016–1147–z.
61. Roriz, C.L.; Barros, L.; Carvalho, A.M.; Santos–Buelga, C.; Ferreira, I.C. *Pterospartum tridentatum*, *Gomphrena globosa* and *Cymbopogon citratus*. A phytochemical study focused on antioxidant compounds. *Food Res. Int.* **2014**, *62*, 684–693. DOI:10.1016/j.foodres.2014.04.036. hdl:10198/10145.
62. Anaswara, M.R.; Suresha, B.S.; Balasubramanian, T.; Sushma, Y.C.; Jaseela, N.M. Medical importance of *Gomphrena globosa* – A systematic review. *Asian J. Pharm. Hea. Sci.* **2022**, *12*(1), 2718–2721. DOI: 10.5530/ajphs.2022.12.18.
63. Yaseen, T.; Slathia, D.; Farooq, I. Improving floral characteristics and yield of globe amaranth (*Gomphrena globosa* L.) through pinching and application of bio fertilizers and its impact on soil fertility. *J. Pharm. Innov.* **2022**, *11*(10S), 425–430. DOI:10.13140/RG.2.2.19073.74087.
64. Gannoun, S.; Mahfoudhi, A.; Flamini, G.; Helal, A.; Mighri, Z. Chemical composition and antimicrobial activities of Tunisian *Salsola vermiculata* L. *J. Chem. Pharm. Res.* **2016**, *8*, 1087–1092.
65. Ribera, A.; Bai, Y.; Wolters, A.M.A.; van Treuren, R.; Kik, C. A review on the genetic resources, domestication and breeding history of spinach (*Spinacia oleracea* L.). *Euphytica* **2020**, *216*(3), 1–21. DOI: 10.1007/s10681–020–02585–y.
